# A Rare Case of Scalp Arteriovenous Malformation in an Adolescent Patient

**DOI:** 10.7759/cureus.37952

**Published:** 2023-04-21

**Authors:** Muna Talafha, Sarah Al Sharie, Osama J Abu-Hamdiyah, Mohammad Araydah, Lou'i Al-Husinat, Giustino Varrassi

**Affiliations:** 1 Medicine, Burnett School of Biomedical Sciences, University of Central Florida (UCF) College of Medicine, Orlando, USA; 2 Clinical Sciences, Yarmouk University, Irbid, JOR; 3 Medicine, Princess Basma Teaching Hospital, Irbid, JOR; 4 Pain Medicine, Paolo Procacci Foundation, Rome, ITA

**Keywords:** avm, arteriovenous malformation, scalp, cirsoid aneurysms, endovascular embolization

## Abstract

Scalp arteriovenous malformations (AVMs) are rare lesions that arise due to a pathological fistulous connection between scalp arterial feeders and draining veins without the involvement of capillary beds. Here, we report a case of a 17-year-old male who presented with an enlarging, pulsatile, mass in the scalp of the parietal region with mild headaches and was diagnosed with a scalp AVM that was treated successfully with endovascular trans-arterial embolization. Scalp AVMs are uncommon extracranial vascular abnormalities that neurosurgeons hardly ever see. To precisely define the angiographic architecture of an AVM and to organize further management, digital subtraction angiography is crucial.

## Introduction

Scalp arteriovenous malformation (AVM), also termed as cirsoid aneurysms were first described by Brecht in 1833 [1]. It is a rare lesion that arises due to a pathological fistulous connection between scalp arterial feeders and draining veins without the involvement of capillary beds and as a result of the abnormal hemodynamics in such an entity, veins enlarge in size and become tortuous leading to the presence of subcutaneous deformation in the scalp [2,3]. Based on how long the anomaly has existed, scalp AVMs can be categorized into either idiopathic or congenital [4]. Scalp AVMs are typically assumed to be congenital, although they have also been documented to occur following infection, and trauma [5,6]. They can occur at any age and have an incidence of 8.1% out of all AVMs [7]. Patients usually present complaining of an enlarging, pulsatile mass, headaches, and tinnitus [8,9]. They can also present with bleeding largely affecting preoperative hemoglobin concentration and requiring preoperative blood transfusion [10].

Lesions are usually detected by physical exam and then confirmed by CT or MRI. However, the best diagnostic tool for this entity is cerebral angiography. Cerebral angiography is useful to delineate the anatomy of scalp AVMs and determine the nature of feeding vessels, and assists surgeons in planning the most suitable and least invasive surgical plan [11]. Because of the differences in high shunt flow, vascular anatomy, and cosmetic deformities, treatment strategies have been documented to be case-dependent, including surgical excision, ligation, or endovascular embolization [2]. Several studies demonstrated different presentations, diagnostic methods, and management options [10,12-14]. However, scalp AVMs remain a rare entity in need of further neurosurgical focus. In this study, we report an interesting, rare case of scalp AVM occurring in an adolescent patient who presented with headaches and a pulsatile mass.

## Case presentation

**Figure 1:**
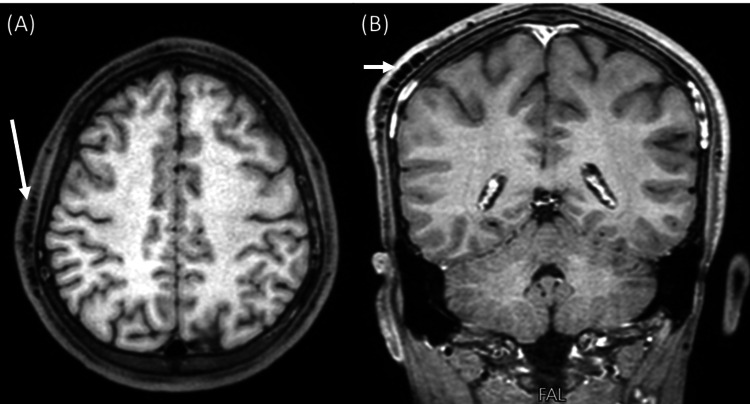
Axial (A) and coronal (B) T1-weighted MRI show tortuous dilated signal void vessels in the scalp of the right parietal region

A 17-year-old male presented to the clinic complaining of a pulsatile right-sided parietal mass for two years, accompanied by mild frequent headaches that were relieved by analgesia. Earlier, the mass did not cause any symptoms and was small in size, but the patient booked his visit when it started to enlarge and when the frequency of headaches increased. There was no history of trauma or infection before the mass. On physical exam, a pulsatile mass with minor hair loss was noticed. The patient had normal motor and sensory functions and cranial nerve examination was unremarkable. His past medical, surgical, and family histories were insignificant. A brain MRI showed tortuous dilated signal void vessels in the scalp of the right parietal region measuring approximately 2.3 cm x 1.7 cm suggestive of a scalp AVM (Figure 1).

**Figure 2:**
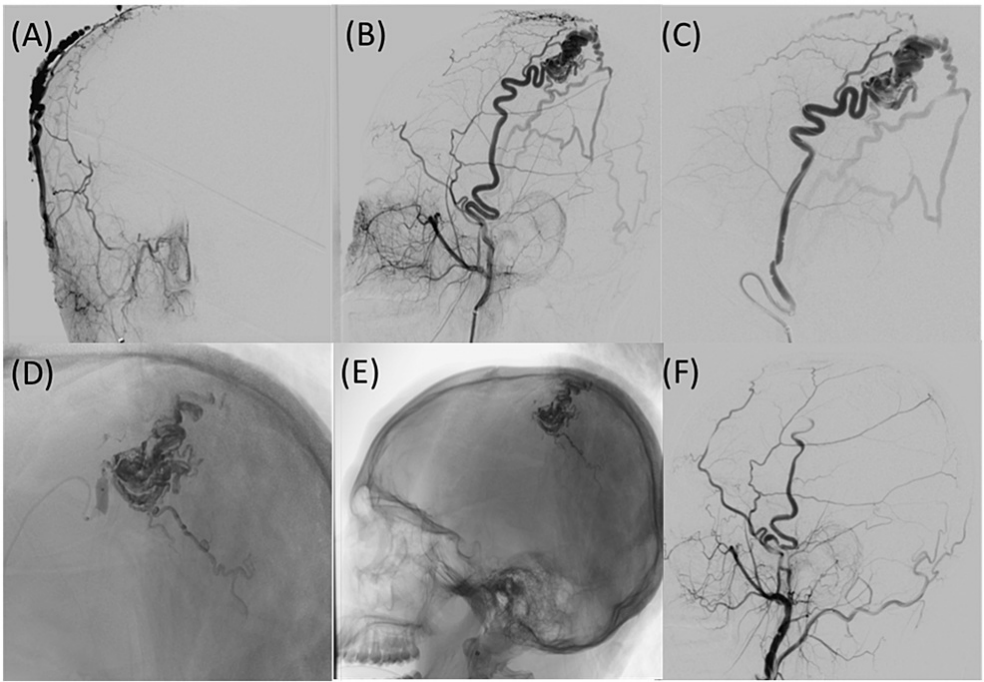
Angiogram of the anterior-posterior external carotid (A), lateral external carotid (B), and lateral superior temporal artery (C) reveal scalp AVM fed by the superior temporal artery. Also observed is the embolic material filling the AVM (D & E), and reaching the venous outflow of AVM. Angiogram of the lateral external carotid post-embolization (F). AVM: Arteriovenous malformation

A right external carotid angiogram was done and confirmed the presence of an AVM receiving supply from the superficial temporal artery, and an endovascular embolization with Onyx 18 (Medtronic, Cork, Ireland) liquid was performed under general anesthesia (Figure 2).

After embolization, the size of the mass instantly decreased, and the pulsation faded. Without any neurologic deficits, the patient experienced an excellent recovery and was sent home. On follow-up after 12 months, the results of the cerebral angiography were negative for the scalp AVM recurrence and no cutaneous necrosis was observed.

## Discussion

Scalp AVMs are uncommon vascular abnormalities with unclear pathogenesis [2,15]. Approximately 10% to 20% of cases of AVMs of the scalp occur after a traumatic head injury [2,16]. The majority of the available literature states that because of the absence of trauma history, scalp AVMs are classified as either congenital or idiopathic [17]. According to some experts, the distinction between congenital and idiopathic AVMs is made based on how long the anomaly has existed i.e., since birth [4].

Multiple hypotheses have been proposed to explain the pathogenesis of non-traumatic scalp AVMs [18]. One hypothesis suggested that scalp AVMs arise from residual, primordial communication between arteries and veins followed by neoangiogenesis. Another hypothesis contended that the existence of vascular hamartomas is responsible for the formation of scalp AVMs. Others suggested that they form when an arteriovenous crosses the scalp which leads to fistula formation [3]. Contemporary experimental models suggested that a prior genetic event may play a role in the development of scalp AVMs [19]. Furthermore, vascular endothelial growth factor (VEGF) has been linked to the development of scalp AVMs, but its exact function is still unclear. Additionally, anomalies in the thickness of the arterial wall, a lack of tight connections and endothelial continuity, and splitting of the elastic lamina have all been linked to AVMs [20].

Depending on where the feeding arteries originate, scalp AVMs are divided into two groups. The first group is called primary scalp AVMs in which the feeding arteries originate from branches of the ophthalmic, external carotid, or vertebral arteries, and the veins drain into the venous system of the scalp. In the second group, scalp AVMs originate from outflow vessels of intracranial vascular anomalies [21].

The clinical manifestations of scalp AVMs are influenced by their size. Lesions initially present as tiny subcutaneous growths and grow into huge, disfiguring masses [8,9]. Other manifestations include throbbing headaches, recurrent hemorrhages, epilepsy, scalp necrosis, congestive heart failure, mental retardation, and intracranial ischemia [22-25]. In some cases, patients may present with pulsatile tinnitus, audible bruit, and palpable thrill due to abnormally increased and turbulent blood flow [25]. Our patient presented with an enlarging pulsatile mass and mild headaches.

After thorough history taking and physical examination, CT, MRI, and angiography are the main steps in the diagnosis of these vascular malformations. Scalp hemangiomas and sinus pericranii are two examples of extracranial vascular pathologies that may be distinguished via vascular imaging [11].

Generally, symptomatic lesions require intervention. Other than for cosmetic reasons, the goal of treating these lesions is to avoid the long-term effects of the ongoing shunting from the arterial system (high pressure) into the venous system (low pressure) [11]. These lesions are challenging to treat due to their multiplex vascular anatomy and the degree of the venous extension in the scalp [17]. Excision of the entire scalp AVM necessitates a thorough understanding of the nature of the malformation including the anatomy of the feeding vessels, draining veins, and the nidus [7]. Given the possibility of cortical venous reflux and the possible risk of venous hypertension, cerebral venous drainage should be evaluated and taken into account when deciding on treatments [11].

Transvenous or transarterial endovascular embolization for managing scalp AVMs has been carried out successfully. Moreover, percutaneous injection of sclerosing agents has been used in selected cases. These procedures also run the risk of side effects such as hair loss, skin necrosis, skin sensitivity, discomfort, and leakage of embolization material into the bloodstream [26]. Our patient did not experience side effects after the embolization was performed. Compared to surgical excision, these techniques are associated with higher rates of recurrence [27,28]. Nevertheless, embolization alone, whether percutaneous or endovascular is still considered an effective technique in managing small-sized AVMs with few feeding vessels [29]. In addition, for patients who are thought to be poor surgical candidates, embolization might be the only treatment option [11].

The most popular technique for treating scalp AVMs permanently is surgical excision [27,30-32]. The main aim is to remove the nidus and the associated galea from the pericranium and the subcutaneous tissue. Dilated arteries and veins of AVMs may extend into the subcutaneous tissue, which can be surgically separated without causing scalp ischemic necrosis [11]. Preoperative embolization may be a useful method to reduce the risk of intraoperative hemorrhage and postoperative recurrence [33]. Modaghegh et al. proposed the use of the double row purse string suture (DROPS) technique in the surgical excision of scalp AVMs to reduce the risk of intraoperative hemorrhage [34]. In our patient, the mass size instantly reduced, and the pulsation faded after endovascular embolization, thus surgical excision was not required.

The most recently published case report of scalp AVM by Briones et al. featured a 49-year-old man who presented to the ER with a giant AVM of the right temporal area accompanied by a large amount of bleeding [10]. The preoperative hemoglobin concentration was 6 g/dL which required a preoperative blood transfusion. Imaging showed numerous feeding arteries with turbulent blood flow arising mainly from three superficial arteries confirming the diagnosis of scalp AVM. Due to the lack of facilities to perform preoperative endovascular embolization, the patient was moved directly to the operating theatre. To substantially minimize blood flow to the AVM, the right external carotid artery was controlled while maintaining a supine position. Then a complete surgical excision with defect reconstruction was performed successfully. The blood loss during the operation was approximately 1.6 L. The significant blood loss that may be encountered in patients with large scalp AVMs highlights the importance of rapid endovascular and surgical interventions.

Shi et al. highlighted the significance and effectiveness of endovascular arterial embolization through a direct percutaneous puncture in the management of scalp AVMs [12]. They presented one case of a frontal scalp AVM and another case of an occipital scalp AVM. In the first case, access to the external carotid artery was approached through an 18-gauge needle direct puncture of the large frontal vein. In the second case, access to the external jugular vein was obtained through the cannulation of the femoral vein. In both cases, embolization was performed successfully with significant resolution of the mass size and pulsation.

In a retrospective case series by Karki (&) Roka, a male predominance in scalp AVMs was observed with a 4:1 male-to-female ratio [13]. The study involved 10 individuals with scalp AVMs in different sites with a mean age of 22.6 years. Approximately two-thirds of the study population (60%) had a history of head trauma and one-third (40%) were spontaneous without trauma history. All patients complained of pulsatile mass and bruit. Throbbing headache was experienced by four patients (40%), and skin ulceration and recurrent bleeding were encountered by three (30%) and one (10%) patients, respectively. The most common location of AVMs were the parieto-occipital (30%) and temporoparietal (30%) areas. All patients were managed with surgical excision without preoperative surgical intervention, and postoperative follow-up was uneventful.

Ung et al. presented an interesting case of a slowly progressing scalp AVM in a 19-year-old male diagnosed four years prior [14]. The patient initially (aged 15) presented with a slowly growing pulsatile mass on the right temporal region without any associated symptoms thus a surgical intervention was not planned and regular follow-up visits were scheduled. The patient was lost to follow-up and then showed up at the age of 19 complaining of severe tension headaches, vision loss, phonophobia, photophobia, tinnitus, and vertigo. Moreover, the substantial growth of the AVM was attributed to head injury as it invaded the occipital region. A diagnostic angiogram was performed and revealed a giant scalp AVM with numerous feeding vessels including the ophthalmic artery ipsilaterally. Preoperatively, to make scalp and facial flaps in preparation for reconstruction of the expected defect after surgical excision, three tissue expanders on the forehead and vertex were placed. Three months afterward, endovascular and surgical interventions with cosmetic reconstruction were successfully performed.

## Conclusions

In this study, we have reported the first case of a scalp AVM in an adolescent patient in Jordan who was treated successfully with endovascular transarterial embolization. Scalp AVMs are uncommon extracranial vascular abnormalities that neurosurgeons hardly ever see. When they result in cosmetic or functional issues, surgical excision is advised. Vascular imaging performed before surgery is essential for identifying AVM morphology and assisting in therapy choices. 
